# miRNA-559 and MTDH as possible diagnostic markers of psoriasis: Role of PTEN/AKT/FOXO pathway in disease pathogenesis

**DOI:** 10.1007/s11010-022-04599-7

**Published:** 2022-11-08

**Authors:** Rana Aldabbas, Olfat G. Shaker, Manal F. Ismail, Nevine Fathy

**Affiliations:** 1 PHD Student at Faculty of Pharmacy, Cairo University, Cairo, Egypt; 2 Medical Biochemistry and Molecular Biology Department, Faculty of Medicine, Cairo University, Cairo, 11562 Egypt; 3 Department of Biochemistry, Faculty of Pharmacy, Cairo University, Kasr El-Aini Street, Cairo, 11562 Egypt

**Keywords:** Psoriasis, Proliferation, miRNA-559, MTDH, PTEN/AKT/FOXO Pathway

## Abstract

Psoriasis is a persistent, inflammatory, autoimmune skin disorder which can be elicited by genetic and environmental factors. Several microRNAs (miRNAs) that are abnormally expressed in psoriasis have emerged as an interesting candidate in psoriasis pathogenesis. However, the expression profile and function of miRNA-559, and its direct target metadherin (MTDH), in psoriasis need to be further illuminated. This study intended to assess miRNA-559 and MTDH levels in skin and sera of psoriatic patients and to investigate their clinical significance in an attempt for developing novel distinct tools for early diagnosis of psoriasis. Moreover, this study aimed at exploring participation of miRNA-559 in regulating MTDH/PTEN/AKT pathway in psoriasis. Expression levels of miRNA-559, AKT, FOXO1 and PTEN were measured by real-time qRT-PCR, whereas MTDH and p27 levels were assessed by ELISA in lesional, non-lesional tissues and serum of 20 psoriatic patients and 20 matching controls. Correlation study was conducted between different parameters. The diagnostic performance of miRNA-559 and MTDH in psoriasis was estimated by receiver operating characteristic (ROC) curve analysis. Expression of miRNA-559 in psoriatic patients was significantly downregulated in both lesional tissues and serum as compared to controls. Conversely, MTDH protein level showed significant increase in both tissues and serum of psoriatic patients and was inversely correlated with miRNA-559 level. Meanwhile, levels of PTEN, AKT and FOXO1 were dramatically changed in psoriatic patients compared to controls. Furthermore, serum miRNA-559 and MTDH displayed comparable diagnostic accuracy in discriminating psoriatic patients from controls. Yet, miRNA-559 demonstrated superior diagnostic performance than MTDH in psoriasis diagnosis. Together, the current findings provide the first suggestion of a new mechanism by which downregulation of miRNA-559 might induce proliferation in psoriasis through modulating PTEN/AKT/FOXO1 pathway by positive regulation of MTDH. Thus, miRNA-559 and MTDH might be proposed as promising diagnostic biomarkers of psoriasis.

**Supplementary Information:**

The online version contains supplementary material available at 10.1007/s11010-022-04599-7.

## Introduction

Psoriasis is a long-lasting, recurrence, inflammatory and proliferative illness which belongs to the group of autoimmune disorders [1]. The etiology of psoriasis is complex and involves various genetic, immunologic and environmental triggers [2]. Chronic plaque-type psoriasis, or psoriasis vulgaris, is the most prevalent kind of psoriasis and is described by distinct areas of pruritic and indurated lesions called plaques [3]. Psoriatic skin lesions are described by hyperproliferation and irregular differentiation of keratinocytes [4]. Yet, the fundamental mechanisms which regulate these cutaneous effects and causes of extreme proliferation and abnormal apoptosis of keratinocytes remain ambiguous [5].

Cell proliferation is one of the key functions of the phosphatidylinositol-3-kinase (PI3K) and protein kinase B (AKT) pathway [6]. The PI3K–AKT–FOXO signaling cascade provides a mean intracellular axis for regulation of cell proliferation [7]. FOXO1 is a component of the forkhead box O (FOXO) transcription factor group [8], which is a target of negative regulation by the PI3K/AKT pathway and is thought to play inhibitory roles on cell proliferation by stimulating cell cycle inhibitors like p27 and p21 [9]. PI3K cascade modulates proliferation of keratinocytes through stimulating AKT and other substrates, as well as inhibiting FOXO [10]. PTEN (phosphatase and tensin homolog) is an important tumor suppressor gene which acts by deactivation of the PI3K/AKT cascade. Previous studies demonstrated that PTEN could be contributed to keratinocytes hyperproliferation associated with psoriasis [11, 12], but its role is still investigated.

MicroRNAs (miRNAs) are a group of tiny endogenous noncoding RNA molecules that play a fundamental role in regulating gene expression [13]. miRNAs associated directly with the 3′-untranslated region (UTR) of their target mRNAs in a base-pairing way and prompt mRNA degradation and/or transcription inhibition [14]. The expression of various mRNAs might, at the same time, be regulated by miRNAs; thus, it is noteworthy that miRNAs modify the expression of 67% of whole human coding genes [15]. Preceding researches have recognized a featured miRNA expression profile in psoriatic skin in comparison to normal skin [16–18].

Numerous of these abnormally expressed miRNAs have been revealed to affect the regulation of keratinocyte proliferation and differentiation [19]. Although miRNA research is an innovative field in dermatology and psoriasis, there is fast growing indication for its chief role in pathogenesis of chronic inflammatory disorders, involving psoriasis and other dermatological conditions [20]. miR-559 acts as a tumor suppressor [21], but the expression profile and molecular function of miR-559 in psoriasis pathogenicity particularly in keratinocyte hyperproliferation need to be further investigated.

Metadherin (MTDH) was previously recognized as a neuropathology-related gene prompted at raised amount in primary human fetal astrocytes, and accordingly, it was named astrocyte elevated gene-1 (AEG-1) [22]. It is a newly discovered oncogene that is upregulated in several kinds of cancers, like neuroblastoma, breast cancer and prostate cancer [23]. MTDH plays major roles in stimulation of a group of signaling pathways, such as PI3K/AKT, Wnt/ β-catenin, NF-κB and MAPK cascades, which play a major role in cancer multiplication, aggression, angiogenesis, metastasis and chemoresistance [24, 25]. AEG-1/MTDH has recently been shown to be associated with regulation of inflammation and immune responses [26]. Nevertheless, the function and biological roles of MTDH in psoriasis have not been clarified. Preceding researches have been covered the contribution of MTDH in the regulation of PTEN/AKT cascade [27, 28]. And it has been recognized to be a direct target of miRNA-559 [21].

Taken together, the aim of the current study was to detect miRNA-559 expression and MTDH levels, not only in lesional and non-lesional tissues, but also in sera of psoriatic patients, and to investigate their biological role in psoriasis. As well, our study targets to shed more light on the participation of miRNA-559 in the MTDH/PTEN/AKT signaling pathway regulation in psoriasis.

## Subjects and methods

This case–control study was carried out at the Medical Biochemistry and Molecular Biology Department, Faculty of Medicine, Cairo University. A written informed consent was obtained from all patients and control subjects. The study protocol was approved by the Research Ethics Committee, Faculty of Pharmacy, Cairo University (BC2622), and conformed to the ethical guidelines of the Helsinki Declaration. The study consisted of 40 Egyptian adult participants who were divided into two groups:

(i) Patients’ group
  It was comprised of 20 patients with psoriasis vulgaris recruited from the Dermatology outpatient clinic, Faculty of Medicine, Cairo University. Patients did not receive any related systemic medications for, at the minimum, four weeks or related topical therapy for not less than two weeks before the beginning of the current study. Patients with erythrodermic or pustular psoriasis, autoimmune diseases, e.g., systemic lupus erythematosus, and patients with hematological or solid malignancies, e.g., leukemia, breast cancer, as well as pregnant and lactating females were excluded.
  Full patient histories were gathered, and all patients were undergone to full clinical examination including: % body surface area affected (% BSA) which was measured by the rule of 9 [29], in addition to, the measurement of severity of psoriasis by psoriasis area and severity index (PASI) score which was estimated for psoriatic patients according to the percent of affected area of the skin and the severity of three clinical signs (erythema, thickness and desquamation) [30]. Besides, for evaluating the biopsied plaque severity score (BPSS) which is altered from Target Plaque Severity Score (TPSS), each lesion was evaluated individually for induration, scaling and erythema depending on a five-point severity scale (0, none; 1, slight; 2, moderate; 3, marked; and 4, very marked), and the scores were added together to yield the TPSS sum score [13-point scale; highest (most severe) score 12] [31].
(ii) Control group
  Twenty age- and sex-matched apparently healthy subjects were involved in this group with no history of chronic dermatological or systemic disease. Preoperative blood samples were collected, and skin biopsies were obtained from extra skin after abdominoplasty, breast reduction or brachioplasty operations.
  For sample collection and storage, a volume of 5 ml of venous blood was withdrawn from each participant in sterile plastic tube, and then, the blood samples were left to clot for 30 min for serum separation by centrifugation at 3000 rpm for 15 min. The separated serum was kept frozen at − 80 °C for further analysis. Two 4-mm punch skin biopsies of lesional and non-lesional skin were obtained from all psoriatic patients and stored at − 80 °C.

### Tissue and serum miRNA-559, AKT, FOXO1 and PTEN assays by RT-qPCR

The extraction of total RNA was done using miRNeasy extraction kit (Qiagen, Valencia, CA, USA) and a QIAzol reagent as reported by manufacturer’s protocol. RNA quantitation and purity measurement for RNA samples were measured by the NanoDrop® (ND)-1000 spectrophotometer (NanoDrop Technologies, Inc., Wilmington, DE, USA).

Reverse transcription (RT) was conducted on extracted RNA in a total amount of 20 μl RT reactions by the miScript II RT kit (Qiagen, Valencia, CA, USA). The RT temperature protocol was as the following: 37 °C for 60 min. and 95 °C for 5 min.

For miRNA-559, RT-qPCR for the mature miRNA, hs-miR-559, was carried out by a miScript miRNA PCR primer assay and a miScript SYBR Green PCR kit (Qiagen, Valencia, CA, and USA) in a final amount of 25 μl per reaction volume using the particular miRNA-559 primers (Cat No MS00010185). Because the endogenous control of miRNA in the serum is unknown, SNORD68 was used as the internal control to normalize the level and for relative quantification of the studied miRNA.

For AKT, FOXO1 and PTEN, expression levels were tested utilizing the miScript SYBR Green PCR kit (Qiagen, Valencia, CA, USA) and GAPDH was used as normalizing endogenous control with customized primers based on the manufacturer’s protocol. The sequences of primer used for AKT, FOXO1 and PTEN are listed in Table 1, and these primers were blasted in the NCBI database to establish their specificity.

**Table 1:** Primer sequences used for qRT-PCR

Gene	Primer sequence
AKT	Forward: 5′-TCT ATG GCG CTG AGA TTG TG-3′ Reverse: 5′-CTT AAT GTG CCC GTC CTT GT-3′ [72]
FOXO1	Forward: 5′- GCT GCA TCC ATG GAC AAC AAC A-3′ Reverse: 5′- CGA GGG CGA AAT GTA CTC CAG TT-3′ [73]
PTEN	Forward: 5′-CAT TGC CTG TGT GTG GTG ATA-3′ Reverse: 5′-AGG TTT CCT CTG GTC CTG GTA-3′ [74]

In-brief, real-time PCR was carried out in 25 μl reaction mixtures where 2.5 μl of properly diluted cDNA template was added to 5 μl of RNase free water, 12.5 μl of miScript SYBR Green PCR Master Mix and 2.5 μl of miScript forward and reverse primers by Rotor gene Q System (Qiagen) with the following conditions: 95 °C for 15 min. then 40 cycles of denaturation at 94 °C for 15 s., annealing at 55 °C for 30 s. and finally extension at 70 °C for 30 s.

Melting curve analysis was used to assess the specificity of PCR products for miRNA-559, AKT, FOXO1 and PTEN. The expression level was evaluated using the Δ*Ct* method, and the calculation of fold change was done by the 2^–ΔΔ*Ct*^.

### Quantitation of MTDH and p27 in tissue and serum using ELISA

Each skin biopsy was weighed, after that each one was homogenized in 300 μl phosphate-buffered saline and then centrifuged at 4000xg for 10 min, and the resulting supernatant was utilized immediately for analysis. Human MTDH and Human p27 ELISA kits provided from Sunlong Biotech (China) were used for determination of MTDH and p27 levels in serum and supernatant.

### Statistical analysis

The statistical analysis was carried out by GraphPad Prism 9.0 statistics software (CA, USA). Expression of qualitative data was in the form of number and percentage, whereas numerical data were described as mea*n* ± SD and median, interquartile range (IQR), or range when suitable. Kolmogorov–Smirnov, D'Agostino and Pearson, and Shapiro–Wilk tests were used in order to test the normality. Analysis of parametric data was done by Student’s t test, while nonparametric data were analyzed by the Mann–Whitney U test or the Kruskal–Wallis test followed by Dunn’s multiple comparisons test as suitable. The *P* value was adjusted for multiple comparisons. Categorical data were analyzed by Fisher exact test. Correlations between different parameters were evaluated by Spearman's rho correlation coefficient. The diagnostic accuracy of the studied parameters was assessed by receiver operating characteristic (ROC) curve, and area under the curve (AUC) was calculated. When AUC < 0.6, it was considered non-significant; furthermore, between 0.7 and 0.89 was regarded as a potential discriminator, while AUC > 0.9 was thought to be a perfect discriminator. *P* value ≤ 0.05 was considered statistically significant. All tests were two-tailed.

## Results

### Demographic and clinical data of psoriatic patients and controls

A total of twenty psoriatic patients (15 males and 5 females) with twenty apparently healthy controls contributed in the current case–control study. The age range in psoriatic patients was 21–66 years with a median age of 36 years and in controls was 22–56 years with a median age of 36 years. There was no significant difference between psoriatic patients and controls relative to age (*P* = 0.932) and gender (*P* = 0.103).

Regarding family history, 16 patients (80%) had positive family history of the disease. Three of the patients (15%) were hypertensive and two of the patients (10%) were diabetic. Demographic and clinical data of the studied subjects including patients and controls are demonstrated in Table 2.

**Table 2:** Demographic and clinical data of study groups

Groups Variables	Control group *N* = 20	Patient group *N* = 20	*P* value
*Age (year)*			
Median (range)	36 (22–56)	36 (21–66)	0.932
*Gender*			
Male, *N* (%)	10 (50)	15 (75)	0.103
Female, *N* (%)	10 (50)	5 (25)	
*Extent of disease (%)*			
Median (range)	–	30 (7–80)	–
Mean ± SD		37.65 ± 22.72	
*PASI Score*			
Median (range)	–	19 (5.6–56.8)	–
Mean ± SD		22.17 ± 12.9	
*BPSS Score*			
Median (range)	–	6 (4–12)	–
Mean ± SD		7.35 ± 2.43	
*Course*			
Progressive, *N* (%)	–	7 (35)	–
Remissions and exacerbations, *N* (%)		13 (65)	
*Duration (months)*			
Range	–	8–360	–
Mean ± SD		122.55 ± 106.91	
*Family history*			
Positive, *N* (%)	–	16 (80)	–
Negative, *N* (%)		4 (20)	
*Hypertension*			
Positive, *N* (%)	–	3 (15)	–
Negative, *N* (%)		17 (85)	
*Diabetes mellitus*			
Positive, *N* (%)	–	2 (10)	–
Negative, *N* (%)		18 (90)	

Data are expressed as mean ± SD, median (range) or number (percentage)

### miRNA-559 expression level in psoriasis

**Fig. 1:**
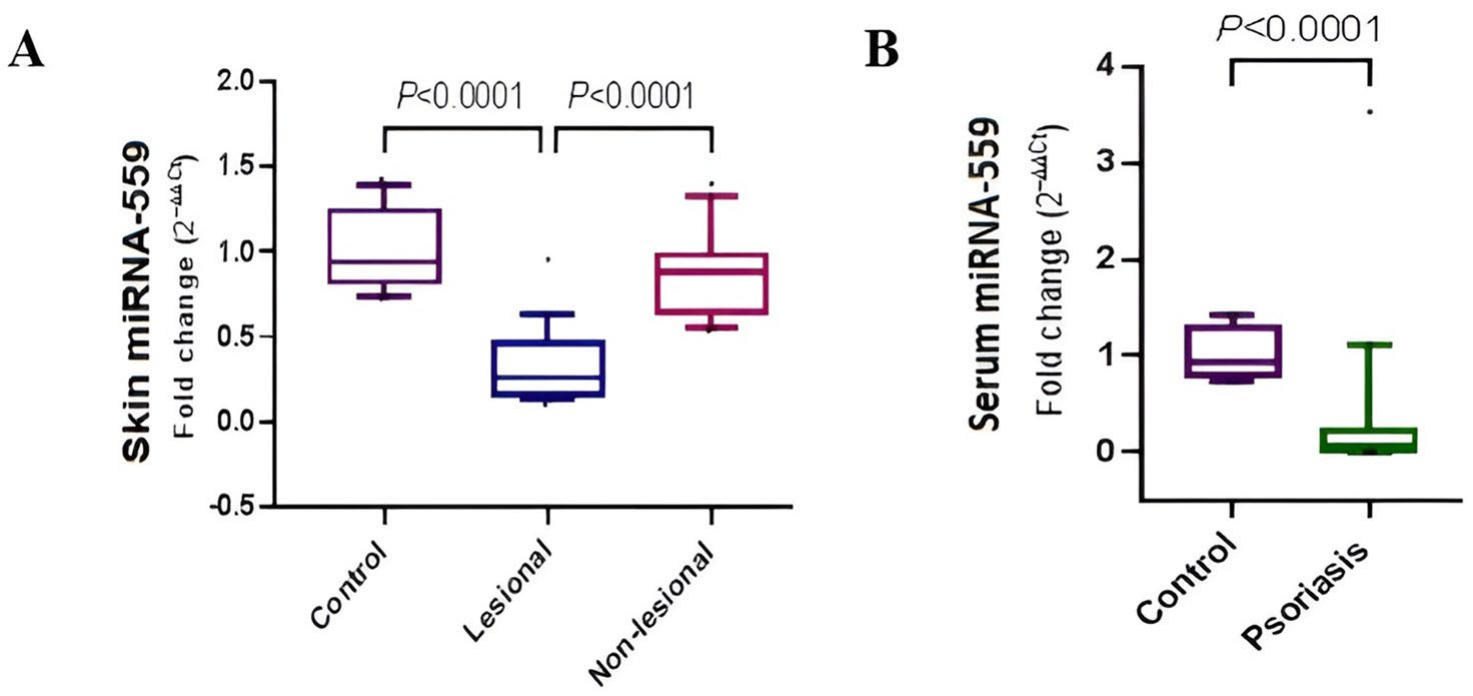
Expression of miRNA-559 in psoriasis. **A** Comparison of skin miRNA-559 expression level between lesional, non-lesional and control. **B** Comparison of serum miRNA-559 expression level between psoriatic patients and controls**.** The box represents the 25–75% percentiles; the line inside the box represents the median and the upper and lower lines representing the 10–90% percentiles. Serum data (two groups) were compared using Mann–Whitney U test. Skin data (three groups) were compared using Kruskal–Wallis test

To examine the role of miRNA-559 in psoriasis, miRNA-559 expression level was measured in skin and sera of both psoriatic patients and controls using qRT-PCR. In skin, expression of miRNA-559 was significantly declined by 3.86-fold in lesional tissues, as compared to controls (*P* < 0.0001). miRNA-559 expression showed no significant difference was found in between non-lessional tissues and controls (*P* = 0.56) (Fig. 1A). Serum miRNA-559 expression among patients was significantly downregulated by 21.2-fold relative to controls (*P* < 0.0001) (Fig. 1B).

### MTDH protein level in psoriasis

To clarify the relation between miRNA-559 and MTDH expression in psoriasis, it was necessary to measure the level of MTDH protein in skin and sera of psoriatic patients by ELISA. MTDH protein level ranged from 247.5 to 378 pg/ml, with a median of 348.6 pg/ml in lesional tissues, and this level was significantly higher than non-lesional tissues (*P* = 0.034) and controls (*P* = 0.04). There was no significant difference in MTDH level between non-lesional tissues and controls (*P* = 0.9) (Table 3). Among patients, MTDH serum level ranged from 225.2 to 1194 pg/ml, with a median of 375.2 pg/ml, and this level was significantly higher than controls (*P* < 0.0001) (Table 4).

**Table 3:** Skin protein levels of MTDH and p27 in study groups

Groups Parameters	Lesional	Non-lesional	Controls	*P* value
MTDH (pg/ml)	348.6 (247.5–378)^a,b^	223 (154.5–321.4)	293 (145.3–348)	0.014
p27 (pg/ml)	519.1 (430–822.3)	590.2 (413.2–1086)	669.6 (564.8–891.6)	0.55

Data are expressed as median (25–75% percentiles)

The presented *P* value is of the nonparametric Kruskal–Wallis test, and *P* value was adjusted for multiple comparisons

^a^significant difference from controls

^b^significant difference from non-lesional

**Table 4:** Serum protein levels of MTDH and p27 in study groups

Groups Parameters	Patients	Controls	*P* value
MTDH (pg/ml)	375.2 (225.2–1194)	149.5 (97.8–188.7)	< 0.0001
p27 (pg/ml)	515 (366.3–694.5)	442.2 (320–499)	0.175

Data are expressed as median (25–75% percentiles), and the presented *P* value is of the nonparametric Mann–Whitney *U* test

### Expression of PTEN/AKT/FOXO1 signaling pathway in psoriasis

**Fig. 2:**
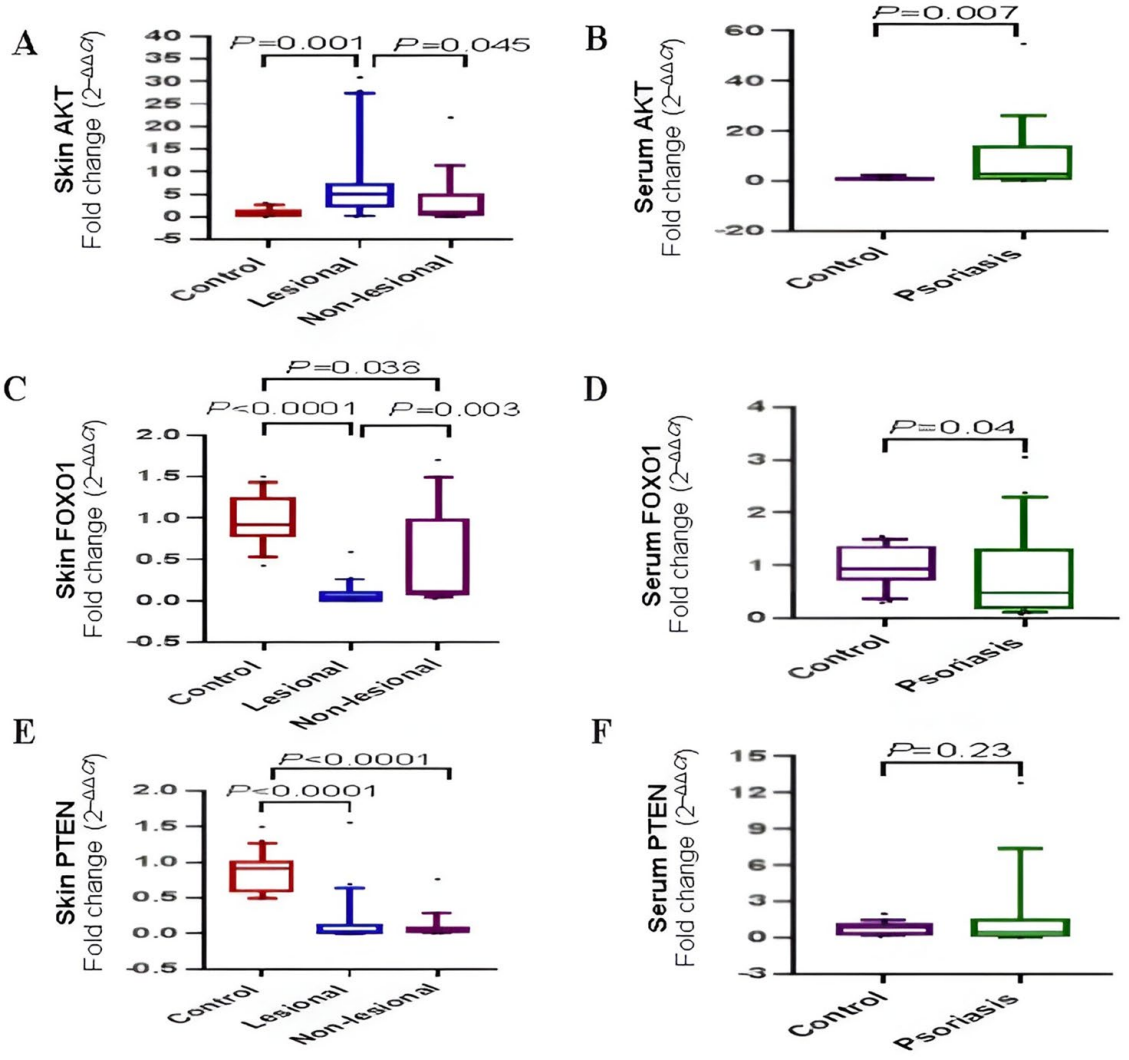
The expression of PTEN/AKT signaling pathway proteins. **A, C** and **E** Comparison of skin AKT/FOXO1/PTEN expression levels between lesional, non-lesional and control. **B, D** and **F** Comparison of serum AKT/FOXO1/PTEN expression levels between psoriatic patients and controls. The box represents the 25–75% percentiles; the line inside the box represents the median and the upper and lower lines representing the 10–90% percentiles. Serum data (two groups) were compared using Mann–Whitney U test. Skin data (three groups) were compared using Kruskal–Wallis test

AKT expression level in lesional tissues was significantly upregulated by 5.014-fold relative to controls (*P* = 0.001) (Fig. 2A). Additionally, serum AKT expression level among patients was upregulated by 2.7-fold, and this value was considered significantly higher than controls (*P* = 0.007) (Fig. 2B).

A significant lower expression level of FOXO1 was found in lesional and non-lesional tissues relative to controls by a median fold change 0.04 (*P* < 0.0001) and 0.11 (*P* = 0.038), respectively. As well, the expression level of FOXO1 in lesional tissues was significantly lower than non-lesional tissues (*P* = 0.003) (Fig. 2C). Among patients, serum FOXO1 expression ranged from 0.21- to 1.29-fold with a median of 0.48 and this value was significantly lower than controls (*P* = 0.04) (Fig. 2D).

PTEN expression level was downregulated in lesional tissues and non-lesional tissues as compared to controls, and the median fold change was 0.032 (*P* < 0.0001) and 0.048 (*P* < 0.0001), respectively (Fig. 2E). Conversely, the current study did not find a significant difference between PTEN expression level in lesional and non-lesional tissues (*P* > 0.99) (Fig. 2E). Serum PTEN expression level did not show a significant difference between patients and controls (*P* = 0.23) (Fig. 2F).

### Protein level of p27 in psoriasis

In lesional tissues, the level of p27 protein ranged from 430 to 822.3 pg/ml, and the median was 519.1 pg/ml. These values were lower than those in non-lesional tissues and controls; however, this decrease did not reach a significant value in both cases (*P* = 1 and 0.8, respectively) (Table 3). Likewise, no significant difference was demonstrated in serum p27 level between patients and controls (*P* = 0.1) (Table 4).

### Correlation study

**Fig. 3:**
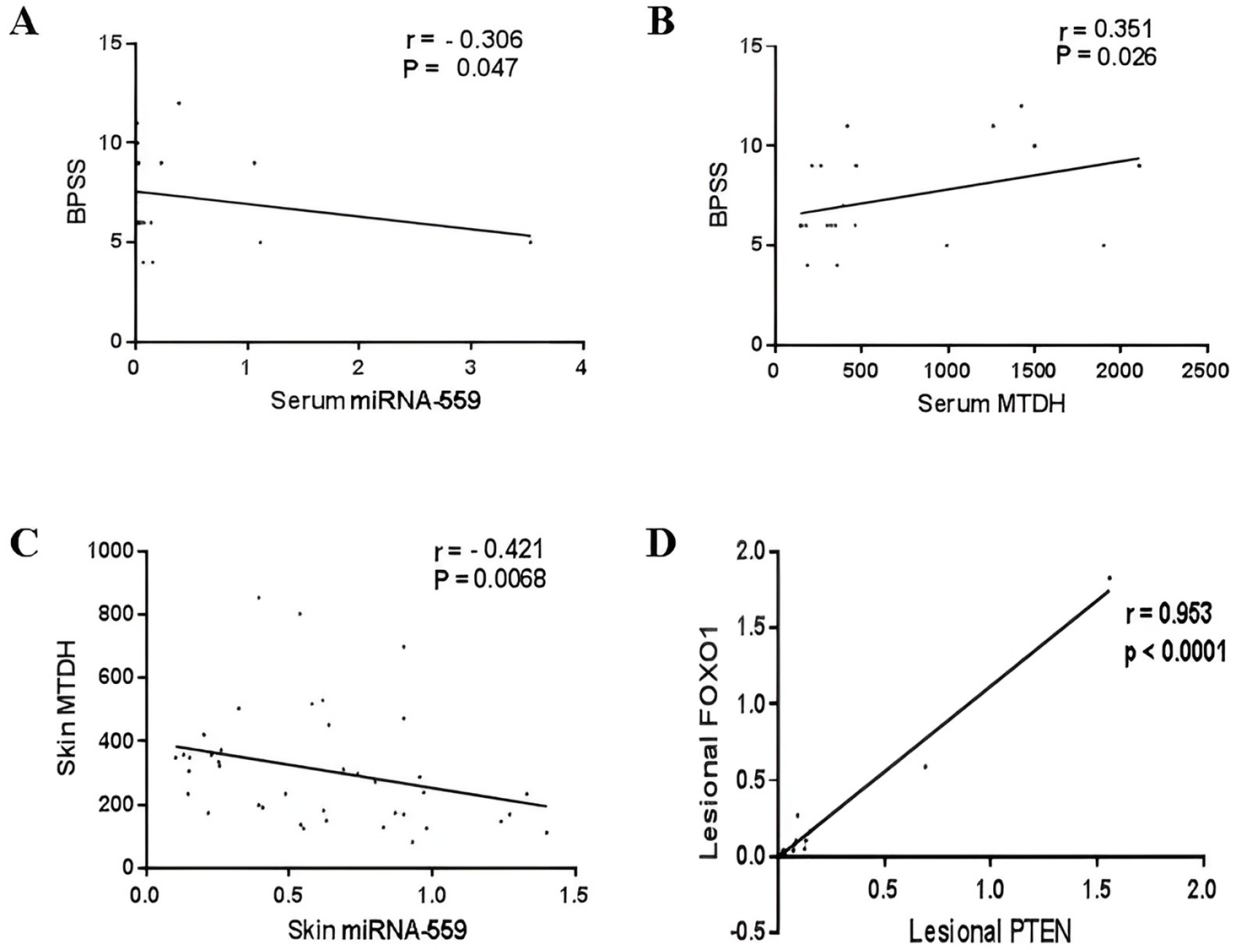
Correlation study. **A** Correlation between BPSS and serum miRNA-559. **B** Correlation between BPSS and serum MTDH. **C** Correlation between skin MTDH and skin miRNA-559. **D** Correlation between lesional FOXO1 and lesional PTEN. Spearman's correlation analysis was used to examine the correlation. *r* = Spearman correlation coefficient

In psoriatic patients, several correlations were found in the whole group patients:

BPSS score was negatively correlated with serum miRNA-559 (*r* = −0.306, *P* = 0.047) (Fig. 3A) and was positively correlated with serum MTDH (*r* = 0.351, *P* = 0.026) (Fig. 3B). In skin, significant negative correlation was observed between miRNA-559 and MTDH (*r* = −0.421, *P* = 0.006) (Fig. 3C), whereas significant positive correlation was found between miRNA-559 and FOXO1 (*r* = 0.332, *P* = 0.036). As well, MTDH was remarkably correlated with AKT (*r* = 0.342, *P* = 0.03). In serum, FOXO1 was positively correlated with each of PTEN (*r* = 0.643, *P* < 0.0001) and p27 (*r* = 0.635, *P* < 0.0001). Moreover, statistically significant positive correlation was detected between PTEN and p27 (*r* = 0.499, *P* = 0.001) (see Supplementary Fig. 1). Another significant positive correlation was detected between skin p27 level and its serum level (*r* = 0.375, *P* = 0.016).

In a subanalysis, several other correlations were found in different sample types (lesional and non-lesional patient groups):

Significant negative correlation was observed between serum miRNA-559 and lesional AKT (*r* = −0.306, *P* = 0.047), whereas serum MTDH was negatively correlated with non-lesional PTEN (*r* = −0.579, *P* = 0.008). In addition, there were significant positive correlations between non-lesional FOXO1 and lesional p27 (*r* = 0.458, *P* = 0.042) (see Supplementary Fig. 2), and between lesional FOXO1 and lesional PTEN (*r* = 0.953, *P* < 0.0001) (Fig. 3D).

### Diagnostic performance of miRNA-559 and MTDH in psoriasis

**Fig. 4:**
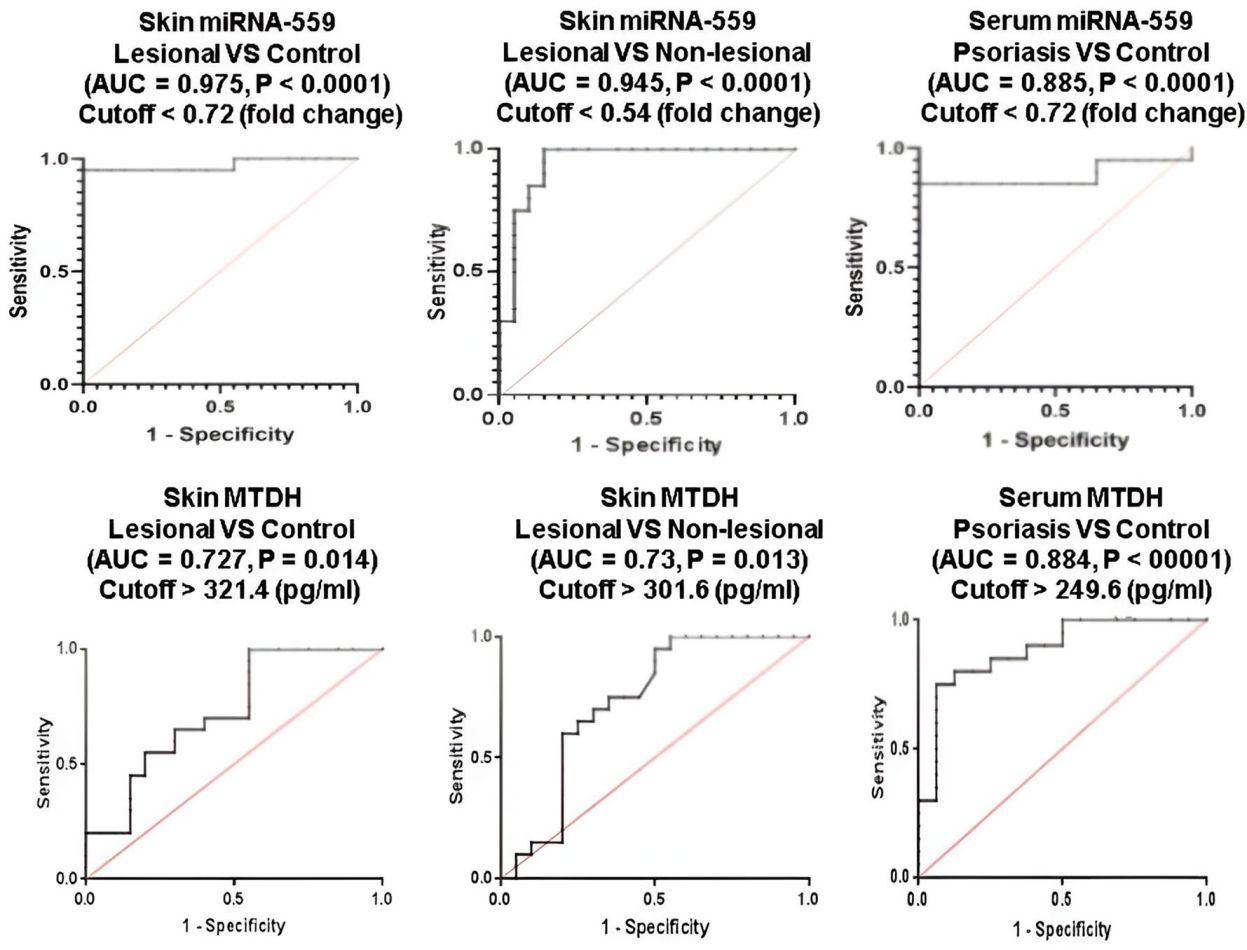
ROC curve analysis of skin and serum miRNA-559 and MTDH

Receiver operating characteristic (ROC) curve analysis for miRNA-559 (Fig. 4) showed that miRNA-559 could be used as a potential marker to distinguish lesional tissues of psoriatic patients from tissues of controls (AUC = 0.975, 95% CI = 0.763–0.997, *P* < 0.0001) with a sensitivity and specificity of 95% at a cutoff < 0.72 (fold) and it also had the ability to discriminate lesional tissues from non-lesional tissues (AUC = 0.945, 95% CI = 0.621–0.967, *P* < 0.0001) with sensitivity of 85% and specificity of 95% at a cutoff < 0.54 (fold). As well, serum miRNA-559 distinguished psoriatic patients from controls (AUC = 0.885, 95% CI = 0.639–0.947, *P* < 0.0001) with a sensitivity and specificity of 85% and 90%, respectively, at a cutoff < 0.72 (fold).

In the same manner, ROC curve analysis for MTDH (Fig. 4) revealed that skin MTDH could differentiate between lesional tissues of psoriatic patients and tissues of controls with sensitivity and specificity of 65% and 70%, respectively (AUC = 0.72, 95% CI = 0.407–0.846, *P* = 0.014), at cutoff > 321.4 pg/ml). MTDH also distinguished lesional tissues from non-lesional tissues (AUC = 0.73, 95% CI = 0.457–0.881, *P* = 0.013) with sensitivity and specificity of 70% at a cutoff > 301.6 pg/ml. In addition, serum MTDH distinguished psoriatic patients from controls (AUC = 0.884, 95% CI = 0.531–0.888, *P* < 0.0001) with a sensitivity and specificity of 75% and 93%, respectively (cutoff > 249.6 pg/ml).

Comparisons of AUC revealed that serum miRNA-559 and MTDH have comparable diagnostic accuracy in discriminating psoriatic patients from controls. Nevertheless, miRNA-559 demonstrated superior diagnostic performance than MTDH in psoriasis diagnosis.

## Discussion

Psoriasis is the most prevalent recurrence inflammatory cutaneous disease caused by contribution of several conditions including genetic factors, environmental triggers and dysregulation of the immune system, which is distinguished by the abnormal interaction between keratinocytes and T cells [32, 33]. Regulating gene expression by miRNA at the post-transcriptional level is thought to be an essential epigenetic process that suggests to be associated with psoriasis pathogenesis [34]. Therefore, clarification of the functions of miRNAs in psoriasis might facilitate the identification of novel molecules for diagnosis and treatment of psoriasis.

Although a lot of miRNAs have been identified, the expression profile and biological role of miRNA-559 in psoriasis have not been reported yet. Herein, the clinical significance of miRNA-559 expression in psoriasis was investigated. In the current study, miRNA-559 expression level was remarkably downregulated in lesional tissues compared to non-lesional tissues and controls. As well, serum expression levels of miRNA-559 were obviously lower in patients in comparison with controls. Likewise, miRNA-559 downregulation was also implicated in human malignances such us glioblastoma [21]. Other studies have reported that miRNA-559 is downregulated in colorectal cancer, and also, it suppresses proliferation of hepatocellular carcinoma in vitro and inhibits cancer aggression of papillary thyroid carcinoma and gastric cancer [35–38]. Furthermore, the notable negative correlation between serum miRNA-559 and BPSS in psoriatic patients indicates that miRNA-559 may have an inhibitory effect on proliferation and progression of psoriatic cells, and it may be suggested to be used as one of the psoriasis severity markers. Taken together, these data indicate that miRNA-559 could act as a promising biomarker for the diagnosis of psoriasis.

The biological role of miRNAs depends on directly regulating the expression of their target genes [39]. MTDH has previously been considered to be a direct target of miRNA-559 through a direct interaction between miRNA-559 and its binding site at 3′UTR of MTDH mRNA leading to mRNA degradation or translation inhibition [21]. MTDH is located on chromosome 8q22 [22] and has been appeared to be extremely expressed in multiple kinds of human malignancy [40–43]. MTDH has been implicated in the tumorigenesis because it regulates several biological roles, including cellular proliferation, cell cycle and apoptosis [25, 44, 45]. In this study, the current findings revealed, for the first time, a remarkable elevation in protein levels of MTDH in both tissues and serum of psoriatic patients. Furthermore, serum MTDH protein level was positively correlated with BPSS. These results explain the function of MTDH in psoriasis pathogenesis, since psoriasis is considered as a proliferative disease which is characterized by extreme proliferation and irregular apoptosis of keratinocytes [5]. Additionally, the inverse correlation between MTDH protein levels and miRNA-559 in psoriatic tissues may suggest that MTDH acts as an immediate target of miR-559 in psoriasis. Determination of the direct target of miR-559 is essential, not only to clarify its functions in psoriasis, but also for the identification of effective novel diagnostic markers for patients with this life threating disease.

Some researches have uncovered the participation of MTDH in the PTEN/PI3K/AKT cascade regulation [46, 47]. In cancers, oncogene Ha-ras was suggested to increase the expression of MTDH by PI3K/AKT pathway. When MTDH is overexpressed, it leads to activation of various oncogenic pathways including PI3K/AKT [48]. Further investigations are required to understand how MTDH can activate PI3K/AKT pathway in psoriasis and identify the precise role played by MTDH in psoriasis progression.

The PI3K/AKT cascade plays a major role in regulation of various cellular functions, such as cell proliferation, repression of apoptosis and cell cycle progression [49]. The participation of the PTEN/AKT cascade in psoriasis has been well recognized [50]. AKT/PKB (also called protein kinase B) is an important target of PI3K and a main regulator of the PI3K cascade. After activation by PI3K through phosphatidylinositol 3-4-5 triphosphate (PIP3), activated AKT translocates from plasma membrane to the cytoplasm and nucleus which induces cell cycle acceleration and cell apoptosis repression [51]. In the present study, the expression of AKT showed significant raise in lesional tissues compared to the non-lesional tissues and controls. The current result is consistent with previous studies [52–54], which revealed that expression of AKT in the keratinocytes in psoriasis lesions was upregulated in comparison with control and non-lesional skin. Additionally, serum level of AKT expression was found, for the first time, to be significantly increased in psoriatic patients relative to controls. Therefore, our results suggest that there might be an association between the raised expression of AKT and the promotion of AKT activity in psoriasis.

Keratinocyte proliferation may be stimulated by activated AKT through phosphorylation of downstream substrates like FOXO [55]. The forkhead box O (FOXO) transcription factor family is recognized to modulate the expression of various genes that are associated with cell proliferation [56]. In our study, expression of FOXO1 was found to be significantly downregulated in serum, as well as in lesional tissues compared to non-lesional tissues and controls. This finding comes in agreement with Liu et al. [55], that demonstrated that the gene expression of FOXO1 is significantly decreased in psoriatic lesions compared with non-lesional and controls. This finding could be attributed to the FOXO1 phosphorylation via AKT which results in FOXO1 nuclear/ cytoplasmic translocation and following degradation by ubiquitin–proteasome system [57]. Therefore, PI3K/AKT/FOXO cascade could contribute in the pathogenicity of psoriasis by downregulation and inactivation of FOXO1 which might lead to enhanced cell proliferation and keratinocyte hyperproliferation.

PI3K/AKT cascade activity is negatively regulated by PTEN [58]. PTEN is a novel tumor suppressor gene that has phosphatase activity towards not only lipids but also proteins [59]. The main tumor-suppressing role of PTEN is to stimulate apoptosis [60], through its action at various points of cell signaling [61]. But the adverse effect of PTEN on PI3K/AKT pathway is the most studied interaction [62]. PTEN dephosphorylates the 3-phosphate on PIP3, the product of PI3K/AKT pathway which is a second messenger for stimulating cell proliferation, to produce PIP2, thereby inhibiting PI3K/AKT pathway and affecting various cell functions including growth and proliferation [63]. Lessened or absent PTEN expression has been recognized in several kinds of tumors, especially glioblastoma, breast cancer and prostate cancer [64]. Malfunction or mutation in PTEN is associated with progress of cancer and also involves in other proliferative disorders such as psoriasis, in which high activity and phosphorylation level of AKT in psoriatic tissues could be related to the PTEN variation [65].

In this research, the expression of PTEN in skin and sera demonstrated significant difference only between lesions and control skin, wherein expression level of PTEN was significantly downregulated in psoriatic lesions compared to control skin, which was in concordance with previous studies [12, 66]. So the current results indicate that stimulation of PI3K/Akt cascade in psoriatic lesions could be aggravated by PTEN downregulation, which leads to extreme proliferation and irregular apoptosis of keratinocytes. On the other hand, PTEN downregulates transcription of cyclin D1, an event which obstructs cell G1-S progression and increases cell cycle stability [67]. Reversely, lower expression of PTEN in psoriatic lesions may cause epidermal propagation by cell cycle accelerating. Interestingly, herein, the study also revealed a significant positive correlation between PTEN and FOXO1 mRNA levels in both lesional tissues and serum. These findings may support the theory that PTEN does not only affect AKT/FOXO pathway but it also acts as a target gene of FOXO, so transcription of PTEN can be enhanced by FOXOs and hyperproliferation of keratinocytes can be arrested [10].

Notably, PI3K/AKT cascade had previously been found to stimulate cell survival by phosphorylating and inhibiting FOXO transcription factor and to affect cellular proliferation by deactivating cell cycle inhibitors like p27 and p21 [68]. In the current study, the aim was to explore the precise molecular mechanism underlying hyperproliferation of keratinocytes in psoriatic patients, so protein level of p27 was measured. However, data did not show any significant difference between p27 protein levels in both skin and sera of both controls and patients. These findings come in accordance with Henri et al. [69], who did not show any statistically significant difference of p27 between psoriatic and control skin. p27 expression may be regulated by several mechanisms, including its subcellular localization, phosphorylation and proteasome-mediated degradation [70]. Moreover, p27 function is not only restricted to cell cycle regulation, but it may also have CDK-independent functions such as regulation of transcription, cellular differentiation, migration and apoptosis [71].

A significant outcome of the current study is that miRNA-559 and its target MTDH were demonstrated as good discriminators between psoriatic patients and controls. Furthermore, important positive correlations were detected in skin between miRNA-559 and FOXO1 and between MTDH and AKT. As well, significant negative correlations were measured between serum miRNA-559 and lesional AKT and between serum MTDH and non-lesional PTEN. These data suggest a novel signaling pathway that links miRNA-559 and its target gene MTDH to cell cycle activation and proliferation which might be essential for detecting innovative diagnostic and therapeutic targets against psoriasis.

Together, these findings demonstrate that downregulation of miRNA-559 in psoriasis may induce proliferation and inhibit apoptosis via PTEN/AKT pathway through positive regulation of MTDH expression. In our study, one limitation is that sample size is relatively small that could, to some degree, decrease statistical power and result in lacking significance of some results, so more extensive population researches are requisite to replicate our results. Even so, we consider that our results are probably emerging implications in diagnosis of psoriasis and may also represent a promising therapeutic approach for the disease.

## Supplementary Information

Below is the link to the electronic supplementary material.

Supplementary file1 (JPG 244 KB)

Supplementary file2 (JPG 175 KB)
