# Supplementary figures and images for: miRNA-559 and MTDH as possible diagnostic markers of psoriasis: Role of PTEN/AKT/FOXO pathway in disease pathogenesis
Source: Mol Cell Biochem. 2022 Nov 8;478(7):1427–38. doi: 10.1007/s11010-022-04599-7 (PMC10209283; doi:10.1007/s11010-022-04599-7)

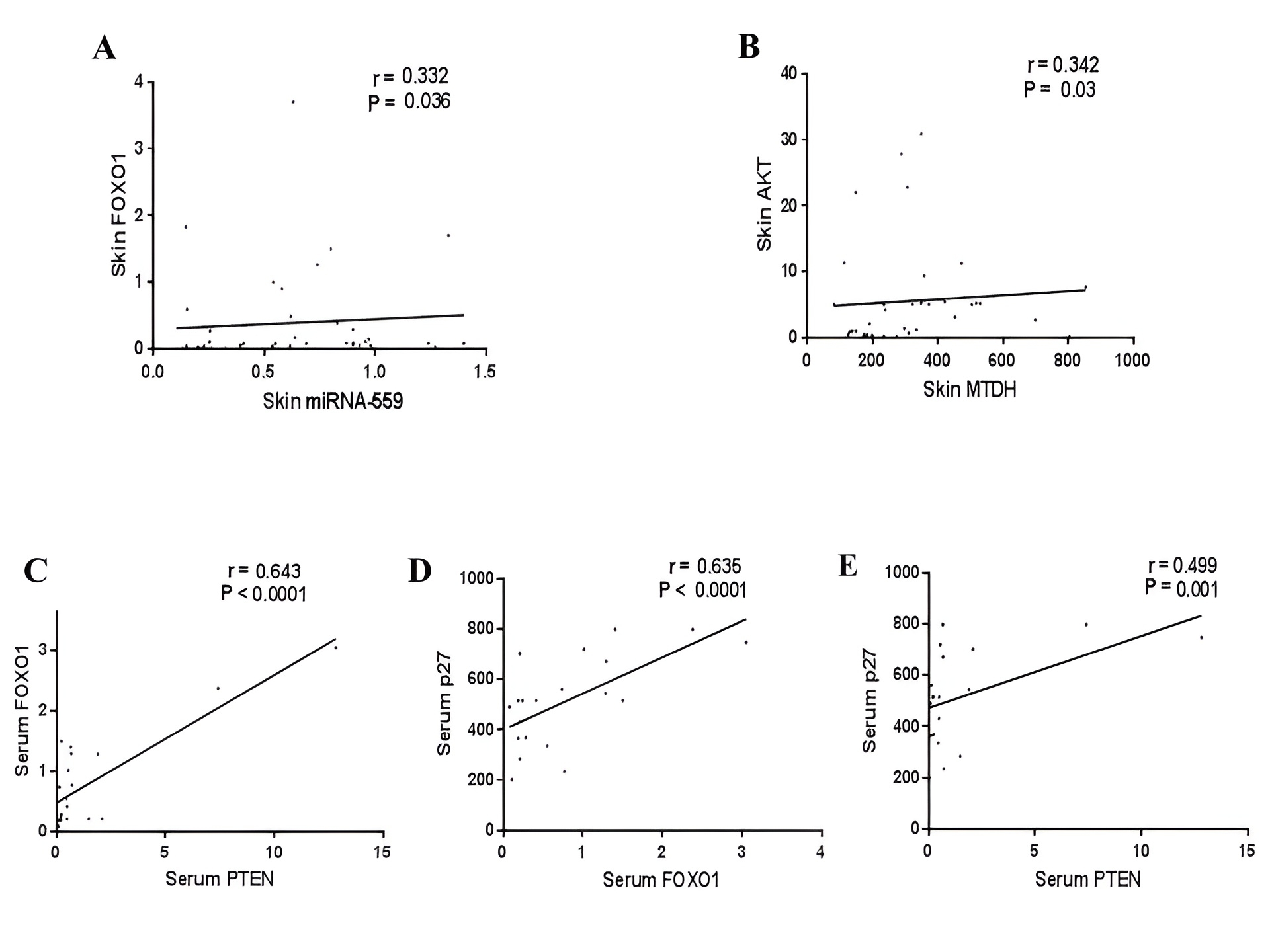

Supplement: Supplementary file 1 — Supplementary file1 (JPG 244 KB) [file 11010_2022_4599_MOESM1_ESM.jpg]

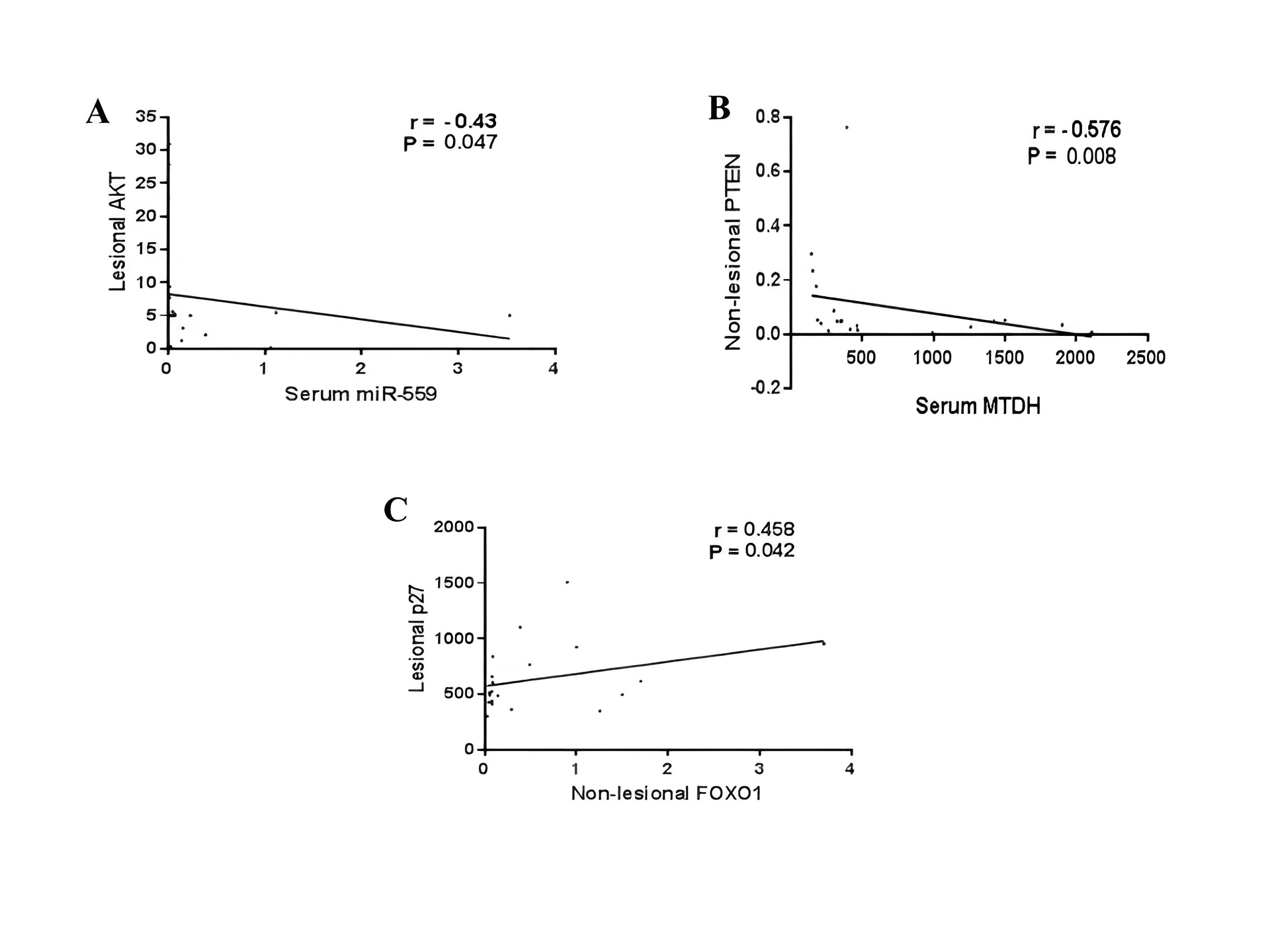

Supplement: Supplementary file 2 — Supplementary file2 (JPG 175 KB) [file 11010_2022_4599_MOESM2_ESM.jpg]
